# A nerve-preserving strategy for endoscopic submucosal dissection of superficial pharyngeal cancers

**DOI:** 10.1055/a-2109-0561

**Published:** 2023-07-13

**Authors:** Takehide Fukuchi, Kingo Hirasawa, Ryosuke Ikeda, Masafumi Nishio, Ryosuke Kobayashi, Chiko Sato, Shin Maeda

**Affiliations:** 1 Division of Endoscopy, Yokohama City University Medical Center, Yokohama, Japan; 2 Department of Gastroenterology, Yokohama City University Graduate School of Medicine, Yokohama, Japan

Endoscopic submucosal dissection (ESD) as a treatment for superficial pharyngeal cancer has been developed and widely accepted by endoscopists in Japan 1 2 . However, a lack of anatomical knowledge can cause complications that are not experienced with gastrointestinal ESD. In the subepithelial layer of the pharyngeal region, the superior laryngeal nerve is a branch of the vagus nerve. The nerve consists of two branches, one of which is the internal laryngeal nerve that supplies sensory fibers to the laryngeal mucosa 3 . Damage to this nerve may result in postoperative dysphagia and hoarseness due to laryngeal hyposensitivity 4 5 . As we have encountered several similar complicated cases, we have developed a strategy for preserving the internal laryngeal nerve during pharyngeal ESD. Here, we present a successful case of nerve preservation ( Video 1 ).

**Video 1** A nerve-preserving strategy for endoscopic submucosal dissection of superficial pharyngeal cancers.


ESD was performed under general anesthesia. Narrow-band imaging (NBI) and Lugol chromoendoscopy clearly revealed the lesion ( Fig. 1 ). We performed a circumferential incision using a dual knife (KD-650Q; Olympus Medical Systems, Tokyo, Japan) from the edge of the aryepiglottic fold. We carefully made a shallow incision to avoid damaging the nerve at the tip of the piriform sinus. After exposing the nerve, we fully injected and continued to dissect the appropriate layer, using the preserved nerve as a landmark ( Fig. 2 ). Finally, we applied multidirectional traction using Fraenkel laryngeal forceps (Nagashima Medical Instruments Co., Ltd., Tokyo, Japan). The lesion was removed en bloc, and the internal laryngeal nerve was completely preserved within 45 minutes without complications ( Fig. 3 ). The tumor was 51 mm in size, and histological examination showed squamous cell carcinoma, negative lateral and vertical margins, and no lymphovascular invasion. No postoperative complications were observed. Distinctive anatomical knowledge and treatment strategies are essential to prevent postoperative complications when performing ESD in the pharyngeal region.

**Fig. 1:**
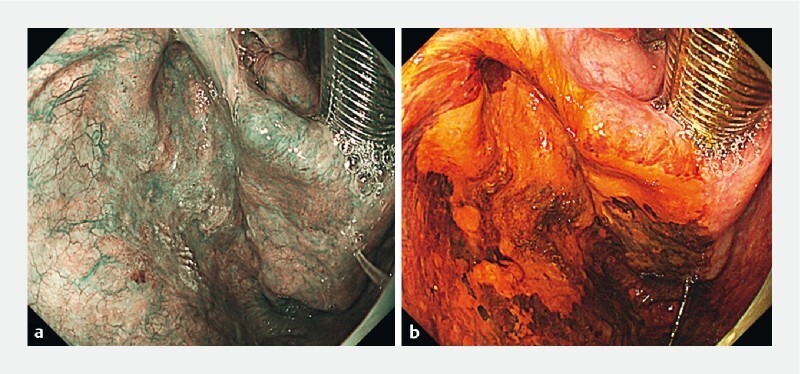
Superficial pharyngeal cancer: endoscopic imaging clearly reveals the lesion. **a** Narrow-band imaging; **b** Lugol chromoendoscopy.

**Fig. 2:**
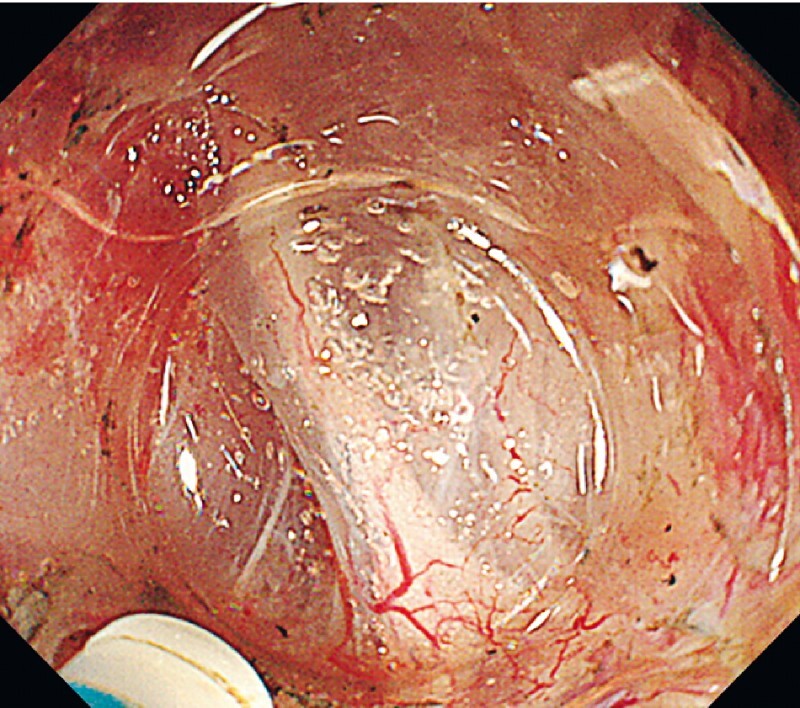
The internal laryngeal nerve is recognizable as a white thick band in the subepithelial layer.

**Fig. 3:**
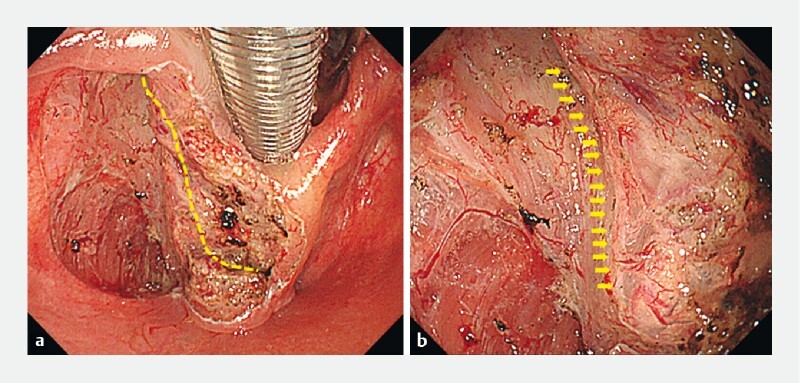
The lesion was removed en bloc, and the internal laryngeal 2anerve was completely preserved. **a** Overview of the line of the nerve; **b** preserved visible nerve.

Endoscopy_UCTN_Code_TTT_1AO_2AG

## Notes

Endoscopy E-Videos

https://eref.thieme.de/e-videos
*E-Videos* is an open access online section of the journal *Endoscopy* , reporting on interesting cases and new techniques in gastroenterological endoscopy. All papers include a high-quality video and are published with a Creative Commons CC-BY license. Endoscopy E-Videos qualify for HINARI discounts and waivers and eligibility is automatically checked during the submission process. We grant 100% waivers to articles whose corresponding authors are based in Group A countries and 50% waivers to those who are based in Group B countries as classified by Research4Life (see: https://www.research4life.org/access/eligibility/ ). This section has its own submission website at https://mc.manuscriptcentral.com/e-videos
